# Geometric morphometrics versus DNA barcoding for the identification of malaria vectors *Anopheles dirus* and *An. baimaii* in the Thai-Cambodia border

**DOI:** 10.1038/s41598-022-17646-6

**Published:** 2022-08-02

**Authors:** Tanawat Chaiphongpachara, Tanasak Changbunjong, Suchada Sumruayphol, Sedthapong Laojun, Nantana Suwandittakul, Kewarin Kuntawong

**Affiliations:** 1 Department of Public Health and Health Promotion, College of Allied Health Sciences, Suan Sunandha Rajabhat University, Bangkok, 10300 Thailand; 2 Department of Pre-Clinic and Applied Animal Science, Faculty of Veterinary Science, Mahidol University, Nakhon Pathom, 73170 Thailand; 3 The Monitoring and Surveillance Center for Zoonotic Diseases in Wildlife and Exotic Animals (MoZWE), Faculty of Veterinary Science, Mahidol University, Nakhon Pathom, 73170 Thailand; 4 Department of Medical Entomology, Faculty of Tropical Medicine, Mahidol University, Bangkok, 10400 Thailand

**Keywords:** Entomology, Biological techniques, DNA sequencing

## Abstract

*Anopheles* (*Cellia*) *dirus* Peyton & Harrison and *Anopheles baimaii* Sallum & Peyton are sibling species within the Dirus complex belonging to the Leucosphyrus group, and have been incriminated as primary vectors of malaria in Thailand. In the present study, DNA barcoding and geometric morphometrics were used to distinguish between *An. dirus* and *An. baimaii* in the international border areas, Trat Province, eastern Thailand. Our results revealed that DNA barcoding based on the cytochrome *c* oxidase subunit I gene could not be used to distinguish *An. dirus* from *An. baimaii*. The overlapping values between intra- and interspecific genetic divergence indicated no barcoding gap present for *An. dirus* and *An. baimaii* (ranging from 0 to 0.99%). However, the results of the geometric morphometric analysis based on the wing shape clearly distinguished *An. dirus* and *An. baimaii*, with 92.42% of specimens assigned to the correct species. We concluded that geometric morphometrics is an effective tool for the correct species identification of these two malaria vectors. Our findings could be used to make entomological surveillance information more accurate, leading to further effective mosquito control planning in Thailand and other countries in Southeast Asia.

## Introduction

Malaria is a mosquito-borne infectious disease caused by unicellular protozoan parasites belonging to the genus *Plasmodium.* It is a significant public health problem in many tropical and subtropical countries^1^. In 2020, the World Health Organization reported an estimated 241 million cases of malaria, leading to 627,000 deaths worldwide^2^. Malaria is endemic in Thailand, especially in the international border areas, because the geographical structure of these areas leads to forest malaria^3,4^. Forest malaria is a term that describes the forest areas associated with malaria transmission or the potential for transmission^5^. Some *Anopheles* mosquitoes (Diptera: Culicidae) are the vectors that transmit *Plasmodium* pathogens to humans via the bite of an infected female *Anopheles*^6^.

Two *Anopheles* species complexes, Dirus and Minimus, are the most important malaria vectors in Thailand^7^. A species complex is a morphologically similar group of organisms, making it difficult to identify their members by gold standard methods based on morphological characteristics^8^. Accurate identification of *Anopheles* species is important in order to understand their true bionomic characters and behavioral traits, facilitating the establishment of crucial guidelines for natural mosquito population control and the protection of communities against malaria^9^.

Malaria is endemic in the Thai–Cambodia border areas^10^. *Anopheles* (*Cellia*) *dirus* (previously known as species A) and *An. baimaii* (previously known as species D) are sibling species within the Dirus complex belonging to the Leucosphyrus group in the Neomyzomyia series^11–13^, and have been incriminated as primary malaria vectors in Thailand and some countries in Southeast Asia^14^. Previously, *An. dirus* was found in sympatry with *An. baimaii* in the Thai–Myanmar and Thai–Cambodia border areas^14,15^. However, these species have very similar morphological and anatomical features, making it difficult to separate the species, and leading to errors in local malaria vector information^16^. The wings of females of the two malaria vector species differ slightly in morphological features. *Anopheles dirus* has a presector dark (PSD) spot on the radius wing vein that extends basally beyond the PSD spot on the costa, reaching the humeral dark of the costa vein or at least beyond the middle of the presector pale spot (PSP) of the costa vein, whereas *An. baimaii* has the PSD spot on the radius vein at the level of the PSD spot on the costa or extending only trivially basally, usually to no more than the middle of the PSP of the costa vein^17^. However, a previous *Anopheles* survey in Kanchanaburi, western Thailand, reported that morphology-based species identification of *An. dirus* and *An. baimaii* is often highly error-prone^18^. Therefore, molecular methods are widely used for identifying *An. dirus* and *An. baimaii*^19^. Multiplex species-specific polymerase chain reaction (PCR) assay based on the internal transcribed spacer 2 region (ITS2) of ribosomal DNA is currently used as the standard molecular method for species identification of sibling members within the Dirus complex^19,20^, but there are several situations in which alternative methods, such as DNA barcoding and geometric morphometrics, are needed.

DNA barcoding is a molecular biology technique commonly used to identify unknown mosquito species, such as specimens which are ambiguous due to damaged morphology caused by field specimen collections^21^. This molecular technique is based on the nucleotide sequences of a standard short DNA fragment (~ 400–800 bp) with low intragroup genetic divergence but high intergroup genetic divergence^22,23^. The cytochrome *c* oxidase subunit I (*COI*) gene is widely used as the universal barcode locus for the identification of mosquito species^24,25^. Several studies have reported the success of DNA barcoding in solving the taxonomic problem of mosquito species identification in many countries^26–28^.

Geometric morphometrics (GM)is a modern morphometric measurement technique developed from measurement standards, which relies on geometric principles to assess size and shape variables for species identification or the study of morphological variations^29,30^. The highlights of the GM technique are that it is inexpensive, rapid, and does not require sophisticated equipment^31^. This technique has been used to distinguish members of groups of insects that are difficult to identify morphologically, such as horse flies (Tabanidae)^32^, stable flies (Muscidae)^33^, muscid flies (Muscidae)^34^, soldier castes (Rhinotermitidae)^35^, and mosquitoes (Culicidae)^30,36,37^. Recently, GM techniques were successful in identifying species members within malaria vector groups in Thailand, including the Maculatus group^38^ and the Minimus complex^39^.

In the present study, DNA barcoding and geometric morphometrics were used to assess the effectiveness of identifying *An. dirus* and *An. baimaii*, sibling species of the Dirus complex, in the international border areas of the Trat Province in eastern Thailand. Before proceeding with alternative methods, the actual species of all samples was verified using multiplex PCR based on the ITS2 region as a gold standard molecular marker for the identification of members of the Dirus complex. The results of this study provide guidelines for the correct identification of two principal malaria vector species that are difficult to distinguish, making entomological surveillance information more accurate, and leading to further effective mosquito control planning.

## Methods

### Ethics statement

Our study was conducted in strict accordance with the guidelines and protocols for animal care and use of the Suan Sunandha Rajabhat University, Thailand. All experimental procedures involving mosquitoes were monitored and approved by the Ethics Committee of the Institutional Animal Care and Use Committee of Suan Sunandha Rajabhat University, Bangkok, Thailand (Approval No. IACOC 64-006/2021).

### Study sites and mosquito collection

**Figure 1:**
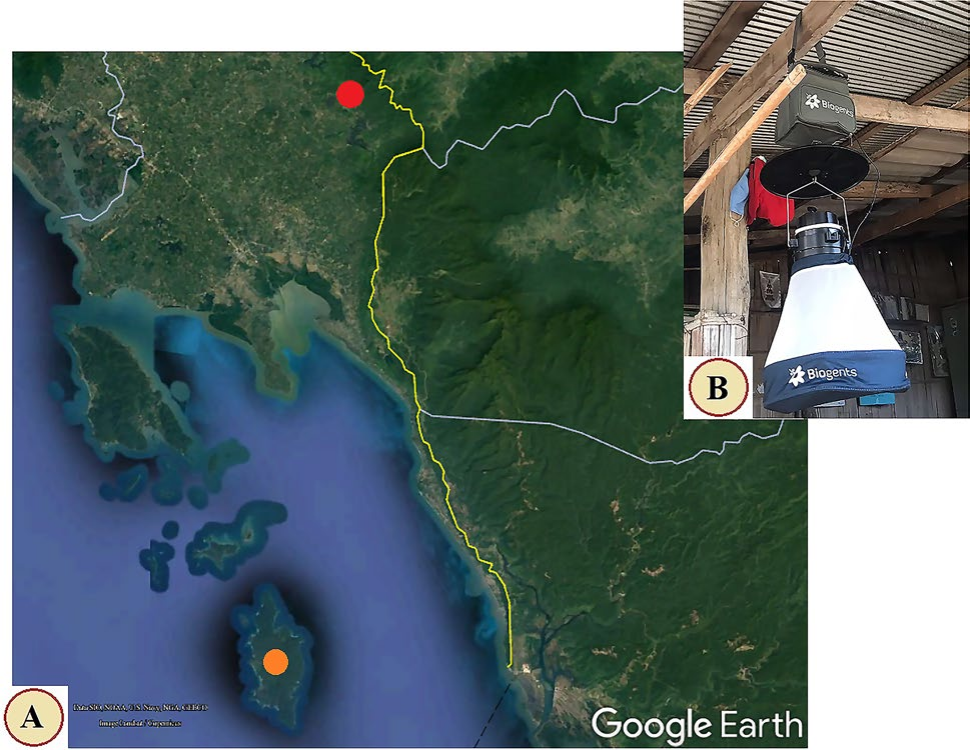
Map of the mosquito collection sites (**A**) including the mainland Thai–Cambodia border (red circle) and the island Thai–Cambodia border (orange circle). This map was generated in Google Earth Pro v 7.1.8 (https://earth.google.com). The BG-Pro CDC-style mosquito trap for the mosquito collecting in this study (**B**).

*Anopheles* mosquitoes were collected from Trat Province, located in the eastern Thai– Cambodia border. Collection was carried out at two sites: the mainland Thai–Cambodia border (12°28′47.2ʺ N 102°40′51.8ʺ E, Dan Chumphon Sub-district, Bo Rai District), and the island Thai–Cambodia border (11°39′21.6ʺ N 102°34′52.6ʺ E, Koh Kood Sub-district, Koh Kood District [Kood island]) (Fig. 1A). Both sites are malaria hot spot areas, according to the malaria report of the Ministry of Public Health of Thailand (accessed at http://malaria.ddc.moph.go.th/). Ten BG-Pro CDC-style mosquito traps (BioGents, Regensbourg, Germany) supplemented with BG-lure cartridges (BioGents) and dry ice (solid carbon dioxide) were used at each border site to catch *Anopheles* mosquitoes (Fig. 1B). The mosquito collection was carried out simultaneously at the two sites over five consecutive nights monthly from 18:00 to 06:00 during June and August 2021. The field-collected mosquitoes were transported back to the laboratory at the College of Allied Health Sciences, Suan Sunandha Rajabhat University, Thailand for species identification. Before the identification of *Anopheles* species, all mosquito specimens were kept individually in 1.5 ml Eppendorf tubes and stored in a freezer at − 20 °C.

### Mosquito identification

*Anopheles* mosquitoes were identified using a morphologic taxonomic key^17^, except for the important malaria vector taxa that are groups and complexes, including the Minimus complex, the Maculatus group, the Dirus complex, and the Barbirostris complex. Member species of the Minimus complex and closely related species (including *An. minimus* sensu stricto [s.s.], *An. harrisoni, An. aconitus, An. varuna* and *An. pampanai*)^40^, the Maculatus group (including *An. maculatus* s.s., *An. pseudowillmori, An. sawadwongporni, An. rampae* and *An. dravidicus*)^41^, and the Dirus complex (including *An. dirus* s.s., *An. scanloni, An. cracens, An. baimaii* and *An. nemophilous*)^42^ were identified and confirmed using the multiplex PCR based on ribosomal DNA ITS2 species-specific primers, following previously published protocols^40–42^. Members of the Barbirostris complex, including *An. barbirostris* s.s., *An. dissidens, An. saeungae, An. wejchoochotei,* and *An. barbirostris* A3 were identified using species-specific primers based on the *COI* gene, according to the protocol proposed by Wilai et al. 2020^43^. Total genomic DNA was extracted from at least two legs of individual adult *Anopheles* specimens using FavorPrep™ Mini Kits (Favorgen Biotech, Ping-Tung, Taiwan) following the manufacturer’s protocols. Platinum Taq DNA polymerase (Invitrogen, Carlsbad, CA, USA) was used in all PCR multiplex assays. PCR amplicons were separated by electrophoresis on 2% agarose gels stained with Midori Green DNA stain (Nippon Gene, Tokyo, Japan) and species-specific bands of DNA were identified using an ImageQuant LAS 500 imager (GE Healthcare Japan Corp., Tokyo, Japan) for true species identification.

### DNA barcoding

To distinguish *An. dirus* and *An. baimaii* using DNA barcoding**,** all PCR reactions of 20 μL contained 1 μL genomic DNA, 1 × reaction buffer, 3 mM MgCl_2_, 0.4 Unit of Platinum Taq DNA polymerase (Invitrogen), 0.2 µM each of forward and reverse primers, 0.2 mM dNTPs, and the remaining volume of distilled water. Universal barcode primer pairs including forward (5'-GGA TTT GGA AAT TGA TTA GTT CCT T-3') and reverse (5'-AAA AAT TTT AAT TCC AGT TGG AAC AGC-3') primers were used to amplify around 707 bp fragments of the *COI* gene^44^, under the following PCR conditions: an initial denaturation at 95 °C for 5 min, followed by five cycles of 94 °C for 40 s, 45 °C for 60 s, 72 °C for 1 min, then 35 cycles of 94 °C for 40 s, 54 °C for 60 s, 72 °C for 1 min, and a final extension of 72 °C for 10 min. Negative controls were included in every PCR round. Amplicons were visualized on 1.5% agarose gels with Midori Green DNA stain using an ImageQuant LAS 500 imager. PCR products with clear DNA bands on agarose gels were sent to Solgent Company, Daejeon, South Korea for DNA sequencing. The forward and reverse *COI* sequence chromatographs obtained were inspected and edited manually using BioEdit software^45^ to generate a consensus sequence for each individual *Anopheles* sample. All consensus sequences of *An. dirus* and *An. baimaii* were compared with those available in the global sequence database to confirm the species, including the NCBI GenBank using the Basic Local Alignment Search Tool (BLAST) and the Barcode of Life Data Systems (BOLD) using the BOLD Identification System. After that the *COI* sequences were automatically aligned using the Clustal X^46^, the genetic distances were computed, including intra- and interspecific genetic divergences based on the Kimura 2-parameter distance model (K2P) and phylogenetic trees constructed using MEGA X software^47^. The maximum likelihood (ML) and neighbor joining (NJ) methods were used for phylogenetic analyses. The node support of the phylogenetic tree was assessed using bootstrap analysis with 1000 replicates. The Tamura 3-parameter with Gamma distribution (G) was the most appropriate nucleotide substitution model for producing the ML phylogenetic tree.

### Wing geometric morphometrics

**Figure 2:**
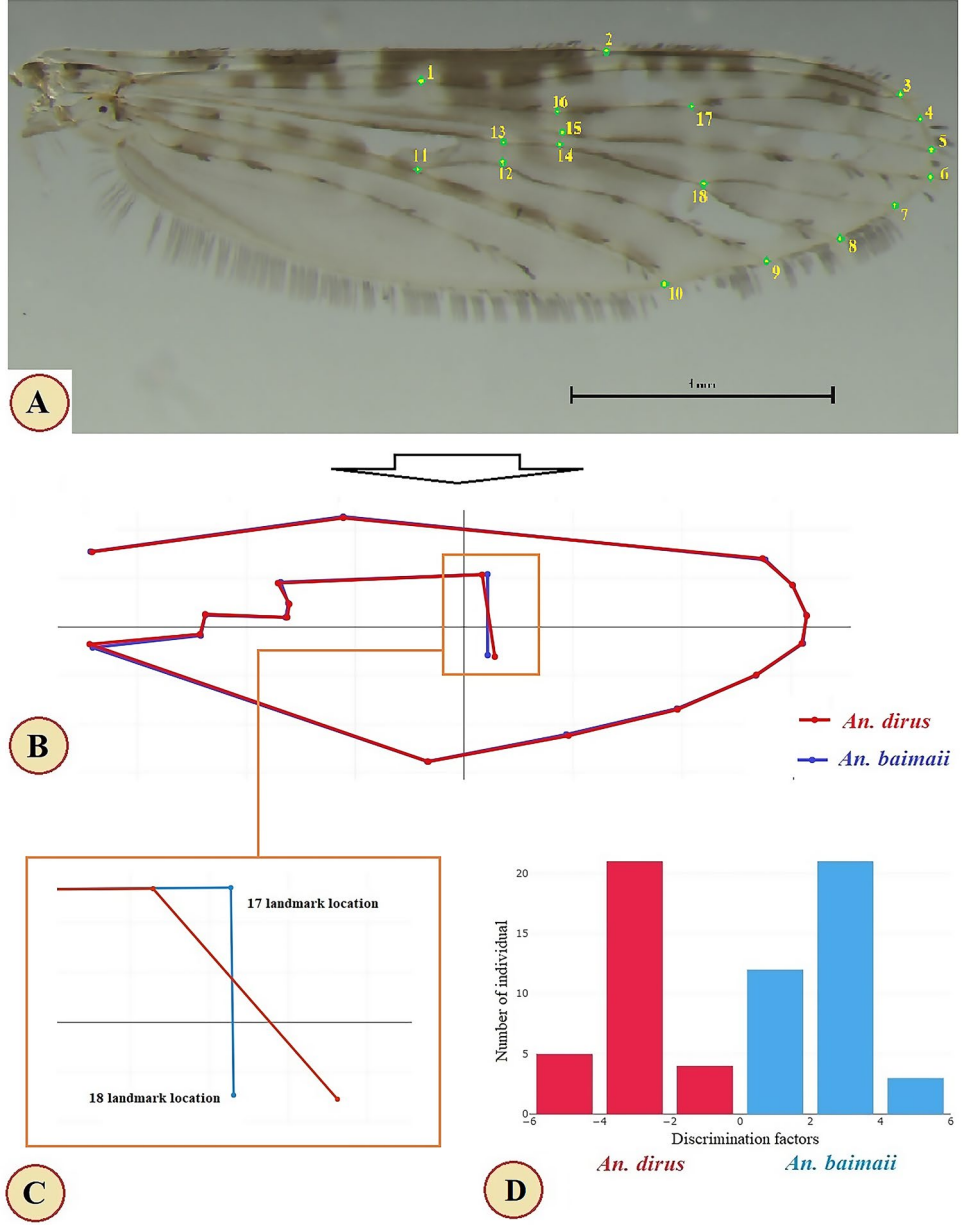
Location of 18 landmarks digitized on the right wing of a female *Anopheles* mosquito (**A**), superposition of the mean landmark configurations (**B**), mean superposition line formed by landmark coordinates at positions 17 and 18 (**C**), and factor map based on discriminant analysis between *Anopheles dirus* (red) and *An. baimaii* (blue) (D).

All complete right wings of female *An. dirus* and *An. baimaii* were detached from the thorax, and the wing scales on the veins were removed and mounted on microscope slides under glass coverslips with Hoyer’s mounting medium. The microscope slides of wings were then photographed using a digital camera attached to a Nikon SMZ 800 N stereo-microscope (Nikon Corp., Tokyo, Japan). Subsequently, the coordinates of 18 landmarks (Fig. 2A) were digitized from each wing image. The landmark locations used in this study were based on previous research studies^48,49^.

XY Online Morphometrics (XYOM)^50^, an online tool, was used for landmark digitization, GM analyses, and graphical outputs in this study. Ten images per species were selected at random and digitized twice by the same person to test the repeatability of the landmarks and evaluate the accuracy of landmark collection in a data set^51^. To assess allometry—the relationship between wing size and wing shape—linear regression of the first (shape derived) discriminant factor on the wing size was performed, and the coefficient of determination was subsequently estimated.

For wing size analyses, the global wing size was estimated from the centroid size (CS), which is defined as the square root of the sum of the squared distances between each landmark and the centroid of the configuration^52^. The CS was calculated after the Procrustes superimposition of the landmark configurations. The wing CS difference between both species was analyzed using one-way ANOVA. The statistical significance of the one-way ANOVA was estimated using a non-parametric procedure (1000 runs) with a threshold of significance of *p*-value < 0.05. Discriminant analysis (or canonical variate analysis) was used to assess wing shape variation among species using a factor map and then the Mahalanobis distance was computed in order to estimate the divergence in shape between the species. The final shape variables were computed using Generalized Procrustes Analysis and used as input to a discriminant analysis. Wing shape differences based on the Mahalanobis distances between *An. dirus* and *An. baimaii* were estimated using a non-parametric procedure (1000 runs) at a *p*-value < 0.05. To assess the correct assignment of *An. dirus* and *An. baimaii* and confirm the reliability of the GM tool when distinguishing between the species based on wing shape, a cross-validated reclassification procedure was carried out based on allocating each individual into the closest group^53^.

## Results

**Figure 3:**
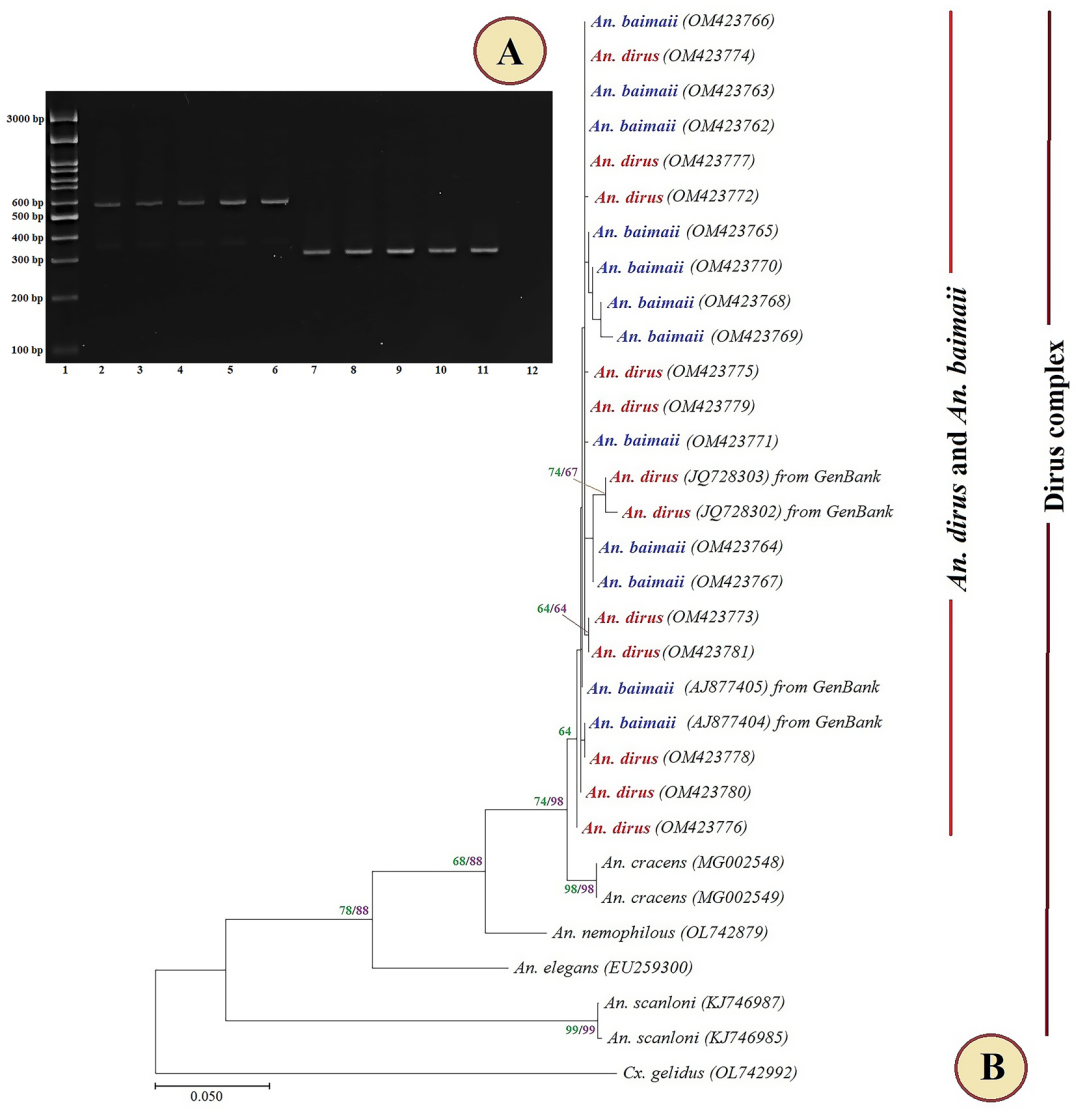
PCR product of the multiplex PCR assay based on the ITS2 region for species identification (**A**). Lane 1: 3000 bp molecular ladder; lanes 2–6: *An. dirus* (562 bp); lanes 7–11: *An. baimaii* (306 bp); lane 12: negative control. Maximum likelihood tree based on the COI sequences of *An. dirus* (red) and *An. baimaii* (blue) in this study, and reference *Anopheles* species sequences obtained from GenBank, with *Culex gelidus* as the outgroup taxon (**B**). Bootstrap values (1000 replicates) of maximum likelihood (green) and neighbor joining (purple) higher than 50% are shown at the nodes.

Two hundred and sixty-four adult *Anopheles* mosquito samples, representing 11 species, of which three species belonged to the subgenus *Anopheles* and eight to the subgenus *Cellia,* were collected from the Thai–Cambodia border areas of Trat Province, Thailand during June and August 2021 (Table 1). Adult *An. jamesii*, *An. philippinensis,* and *An. pseudojamesi* specimens were morphologically identified. Other *Anopheles* mosquitoes were identified to the species using multiplex PCR. Ten *Anopheles* spp. were collected from the mainland border with the exception of *An. baimaii*, which was found in the island border (16.29% of the total *Anopheles* captured in this study). The PCR product of the multiplex PCR assay for the identification of member species in the Dirus complex is shown in Fig. 3A.

**Table 1:** *Anopheles* species and numbers collected in the Thai–Cambodia border areas, Trat Province, Thailand, and identification methods used.

*Anopheles* spp.	Number of *Anopheles* (%)		Identification methods
	Mainland border	Island border	
Subgenus *Anopheles*			
*An. dissidens*	19 (8.60)	–	Multiplex PCR
*An. saeungae*	16 (7.24)	–	Multiplex PCR
*An. wejchoochotei*	77 (34.85)	–	Multiplex PCR
Subgenus *Cellia*			
*An. aconitus*	17 (7.69)	–	Multiplex PCR
*An. baimaii*	–	43 (100)	Multiplex PCR
*An. dirus* s.s	41 (18.55)	–	Multiplex PCR
*An. jamesii*	9 (4.07)	–	Morphology
*An. maculatus* s.s	7 (3.17)	–	Multiplex PCR
*An. minimus* s.s	20 (9.05)	–	Multiplex PCR
*An. philippinensis*	9 (4.07)	–	Morphology
*An. pseudojamesi*	6 (2.71)	–	Morphology
Total	221 (100)	43 (100)

### DNA barcoding for species identification

After confirmation of the true species, partial sequences of the *COI* gene were amplified using universal barcode primers from ten randomly selected specimens of *An. dirus* and *An. baimaii.* No insertions, deletions, stop codons, or pseudogenes were found in any of the *COI* sequences, when the chromatograms were checked. The average nucleotide composition percentages of the *COI* sequences of *An. dirus* and *An. baimaii* were A = 30.3%, T = 39%, C = 15%, and G = 15.7%, and the sequences were AT rich, with an average AT content of 69.3%, higher than the GC content (30.7%). The average intraspecific genetic divergence percentages of *An. dirus* was 0.51%, ranging from 0% to 1.14%, and that of *An. baimaii* was 0.35%, ranging from 0% to 0.71%. The average interspecific divergence between the species was 0.47%, ranging from 0% to 0.99%. Thus, the intra- and interspecific divergence showed overlaps between the two *Anopheles* spp., indicating no barcoding gap. For identification based on the online genetic database, the *COI* sequences of *An. dirus* and *An. baimaii* from this study were compared with those available in the NCBI using BLAST, and in the BOLD database and were shown to have identity overlaps between the species.

Our *COI* sequences in this study, and several sequences available from GenBank, were used for construction of a phylogenetic tree (Table 2). The ML tree showed the phylogenetic relationships of the members of the Dirus complex (Fig. 3B). All sequences of *An. dirus* and *An. baimaii* from this study and GenBank were unambiguously assigned to the same clade. Other members of the Dirus complex, including *An. cracens, An. nemophilous*, *An. elegans,* and *An. scanloni* showed a clear-cut separation between the species. *Culex gelidus,* as an outgroup species, was clearly separated from clusters of species members within the Dirus complex.

**Table 2:** GenBank accession numbers of the *COI* of *Anopheles dirus* and *An. baimaii* sequences in this study and reference mosquito species used for construction of the phylogenetic tree.

*Anopheles* spp.	Locality	Sequence source	GenBank accession no
*An. dirus*	Thailand: Trat	In this study	OM423772-81
*An. dirus*	China: middle Hainan	GenBank	JQ728302-03
*An. baimaii*	Thailand: Trat	In this study	OM423762-71
*An. baimaii*	Thailand: Phang Nga	GenBank	AJ877404-05
*An. cracens*	Malaysia: Pahang, Sg Ular	GenBank	MG002548-49
*An. nemophilous*	Thailand: Ratchaburi	GenBank	OL742879
*An. elegans*	India: Wynad, Kerala	GenBank	EU259300
*An. scanloni*	Vietnam	GenBank	KJ746985
*An. scanloni*	Vietnam	GenBank	KJ746987
*Cx. gelidus* (out group)	Thailand: Chiang Mai	GenBank	OL742992

### Geometric morphometrics for species identification

**Figure 4:**
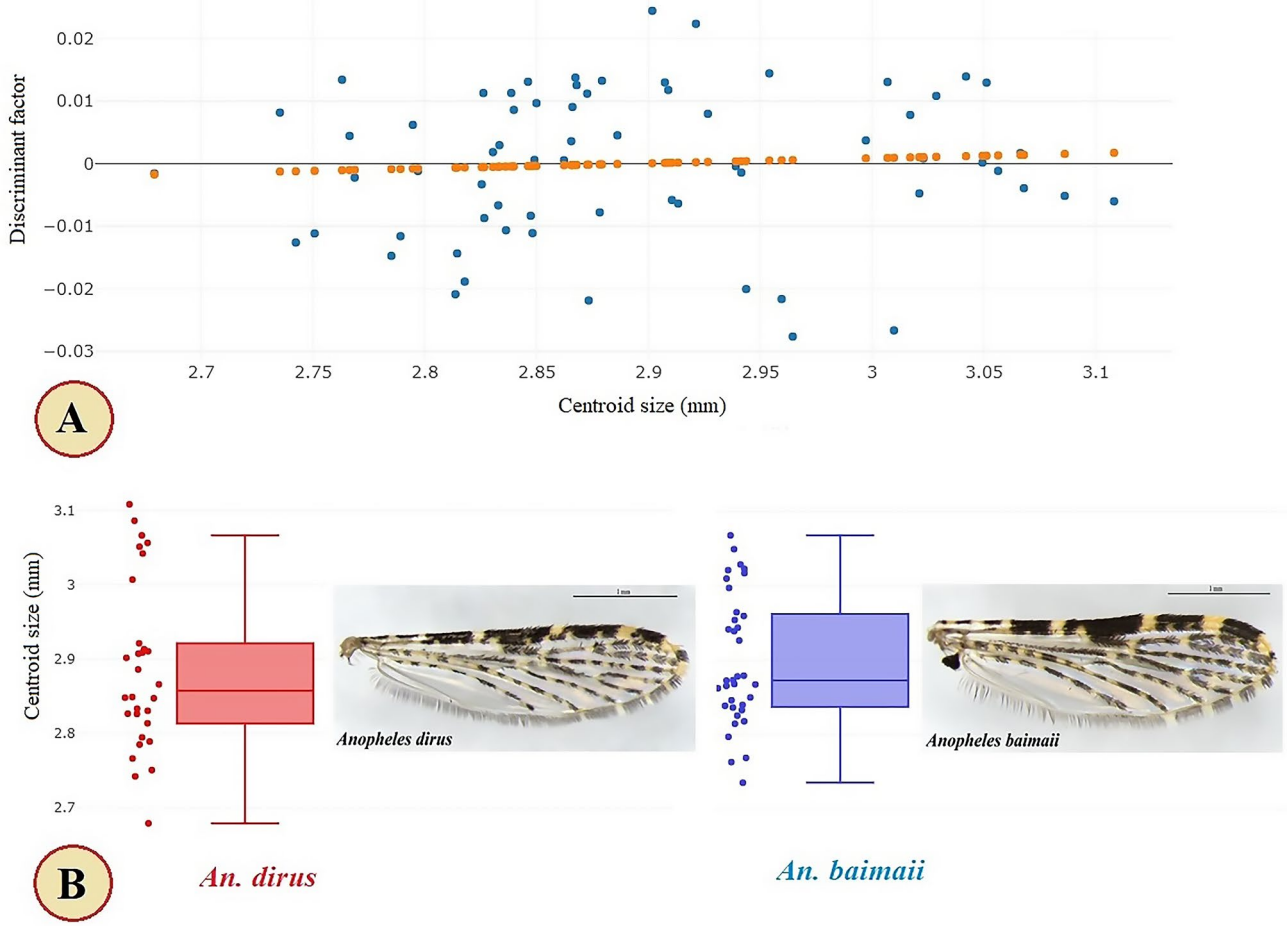
Scatter plot showing the linear regression prediction (orange dotted line) for examining the allometric effect (**A**), and boxplots showing the variation of wing centroid sizes (CS) of *Anopheles dirus* (red) and *An. baimaii* (blue) (**B**). Each quartile box displays the median as a vertical line across the middle and the quartiles indicating the 25th and 75th percentiles at its ends.

Thirty wings from *An. dirus* and thirty-six wings from *An. baimaii* were used for GM analyses. The precision and accuracy of the digitization of landmarks in the wing image sets showed good scores, based on their repeatability. The repeatability scores were more than 95% for size and shape. Assessment of the allometric effect showed that our sample exhibited no apparent relationship between size and shape (r^2^ = 0.2%), which was apparent from the relatively straight line of the linear regression (Fig. 4A).

The wing CS variations of *An. dirus* and *An. baimaii* are shown in Fig. 4B. The mean wing CS of *An. dirus* was 2.89 mm, ranging from 2.68–3.11 mm, while *An. baimaii* was 2.90 mm, ranging from 2.74–3.07 mm (Table 3). No significant difference in wing CS between species was found (*p* > 0.05).

**Table 3:** Mean wing centroid size of *Anopheles dirus* and *An. baimaii,* and statistical characteristics*.*

*Anopheles* spp.	n	Mean	Variance	Standard deviation	*p*-value
*An. dirus*	30	2.89	0.01	0.11	0.60
*An. baimaii*	36	2.90	0.01	0.09	

For wing shape analyses, visualizations of the wing shapes from the superposition of the mean landmark configurations were created before the extraction of the final shape variables (Fig. 2B). The superimposed landmark coordinates at positions 17 and 18 varied the most between *An. dirus* and *An. baimaii* (Fig. 2C). The factor map based on discriminant analysis of the wing shape showed that no samples of the species overlapped (Fig. 2D). The Mahalanobis distance between the species was 5.86, and was statistically significant (*p* < 0.05). These results showed that the wing shapes of *An. dirus* and *An. baimaii* were clearly different. A pairwise cross-validated reclassification test showed excellent accuracy scores for correct identification, ranging from 91.67 to 93.33% (Table 4).

**Table 4:** Cross-validated scores based on the wing shapes of *Anopheles dirus* and *An. baimaii*.

*Anopheles* spp.	Percentages of correctly assigned species	Assigned/Observed (individuals)
*An. dirus*	91.67%	28/30
*An. baimaii*	93.33%	33/36
Total performance	92.42%	61/66

## Discussion

A recent study by Pimnon and Bhumiratana (2018) in the Thai–Cambodia border area of Trat Province, Thailand^10^ investigated the distribution of six *Anopheles* spp.: *An. dirus, An. minimus, An. pseudowillmori, An. campestris, An. barbirostris,* and *An. jamesii*. However, in this study, 11 *Anopheles* spp., including *An. dirus, An. aconitus, An. baimaii, An. jamesii, An. minimus, An. dissidens, An. maculatus, An. philippinensis, An. pseudojamesi, An. saeungae,* and *An. wejchoochotei*, were collected from the Thai–Cambodia border. An increasing number of *Anopheles* spp. were reported in this survey, due to the use of molecular biology techniques in the investigation. In particular, sibling members within the *Anopheles* species complex are difficult to identify species by standard morphological methods^43,54^. In the present study, three sibling species of the Barbirostris complex, *An. dissidens, An. saeungae*, and *An. wejchoochotei*, and two sibling species of the Dirus complex, *An. dirus* and *An. baimaii*, were identified using species-specific multiplex PCR. This study is the first time that *An. baimaii* was found in an island area, Thailand. This *Anopheles* spp. has been reported to have a distribution restricted to some forested areas of central, southern and western Thailand, especially the Thai–Myanmar border^14^.

In Thailand, five species of the Dirus complex are reported: *An. dirus* (species A), *An. cracens* (species B), *An. scanloni* (species C), *An. baimaii* (species D), and *An. nemophilous* (species F)^9,13,16^. However, only *An. baimaii* and *An. dirus* are considered as the primary vectors of malaria in Thailand, and can be found along the Thai–Cambodia border. Tananchai et al. 2012^16^ studied the biology of two *Anopheles* spp. in Pu Teuy Village, Kanchanaburi Province, western Thailand, and found that they had different patterns of feeding activity. Their study was the starting point for understanding the behavioral differences between the two vector species, and formed the basis of strategies for malaria control and prevention in areas in which malaria is endemic. However, a variety of effective alternative techniques are needed to aid in the identification of both species, according to the appropriate situation.

DNA barcoding is widely recognized as a modern molecular technique which can help identify unknown mosquito specimens to the species level^55^. However, the results of this study revealed that DNA barcoding based on the *COI* gene, as the “gold standard” barcode for animals^24,25^, could not be used to discriminate *An. dirus* from *An. baimaii*. The overlapping values for intra- and interspecific genetic divergence indicated that there was no barcoding gap for *An. dirus* and *An. baimaii*. The lack of a barcoding gap means that the genetic distances of our *COI* sequences cannot be used to identify *An. dirus* and *An. baimaii*, due to insufficient nucleotide differences making it impossible to delineate species boundaries^56^. This finding was consistent with phylogenetic analysis based on the *COI* sequences, which found that the species were not grouped separately into clades, while the other members of the Dirus complex, including *An. cracens, An. nemophilous*, *An. elegans*, and *An. scanloni* were clearly separated. Therefore, although this technique cannot separate *An. dirus* and *An. baimaii*, it can effectively classify the other species members of the Dirus complex. Our results are consistent with previous studies reporting that partial *COI* gene sequences failed in distinguishing between *An. dirus* and *An. baimaii* but can clearly distinguish other species of the Dirus complex and the Leucosphyrus group^57^. Often, this technique has failed to identify members in species complexes or closely related species, due to overlapping intra- and interspecific variation caused by incomplete lineage sorting between recently diverged species, such as some member species in the *Anopheles strodei* subgroup^58^.

In contrast, the results of GM based on wing shape analysis revealed an excellent differentiation between *An. dirus* and *An. baimaii*, with 92.42% of specimens correctly assigned based on a cross-validated reclassification procedure. The discriminant analysis identified the differences between the wing shapes of *An. dirus* and *An. baimaii.* Visual wing shape based on superposition of the mean landmark configurations showed an incompatibility between *An. dirus* and *An. baimaii* at landmark positions 17 and 18. These results suggest that the wing venation of each species is unique, leading to the successful identification based on wing GM analyses. The shape of the wing was recognized as a valuable variable for GM analyses in identifying mosquito species due to its stable character and distinctive structure^31,36^. These findings are in agreement with previous studies which found that GM techniques based on wing shape analyses can assist in the identification of members among the *Anopheles* species group and complex in Thailand, such as two species of the Minimus complex, *An. minimus* and *An. harrisoni*^39^, and three species of the Maculatus group, *An. maculatus, An. sawadwongporni*, and *An. pseudowillmori*^38^. Wing size is a variable that is not suitable for species identification by GM because it varies according to environmental influences^59,60^. Our analyses of the wing size based on CS in this study indicated that *An. dirus* and *An. baimaii* were not statistically different. Consistent with the results of assessing allometry, the size and shape of the wings had no relationship. Therefore, the wing size of *An. dirus* and *An. baimaii* was not used for species identification.

## Conclusions

The findings of this study revealed that the GM technique can be used to effectively distinguish *An. dirus* from *An. baimaii*. This modern technique is valuable in laboratories with budget or molecular biology equipment constraints. However, the use of the GM technique should be revalidated each time in every other target area to prevent errors from morphological variations of mosquito specimens in response to environmental factors. In addition, further studies with a large number of specimens are needed to increase the confidence in this technique. For molecular techniques, our results show that DNA barcoding cannot distinguish *An. dirus* from *An. baimaii* due to the low difference in the nucleotide sequences of the *COI* gene of the species. However, the multiplex PCR method based on ITS2 remains the most effective in identifying both species. This study is an important guideline for identifying *An. dirus* and *An. baimaii* sibling species within the Dirus complex. These species are important malaria vectors in the border areas, and the ability to identify them will lead to the development of further effective strategies for the control of the *Anopheles* population in Thailand and other countries in Southeast Asia.

## Data Availability

All DNA barcode sequences of *An. dirus* and *An. baimaii* were submitted and available in the GeneBank Database (https://www.ncbi.nlm.nih.gov/nuccore), accession numbers: OM423762- OM423781 (Table 2).
